# *Supporting Parents & Kids Through Lockdown Experiences (SPARKLE)*: A digital parenting support app implemented in an ongoing general population cohort study during the COVID-19 pandemic: A structured summary of a study protocol for a randomised controlled trial

**DOI:** 10.1186/s13063-021-05226-4

**Published:** 2021-04-10

**Authors:** Katarzyna Kostyrka-Allchorne, Cathy Creswell, Sarah Byford, Crispin Day, Kimberley Goldsmith, Marta Koch, Walter Muruet Gutierrez, Melanie Palmer, Jasmine Raw, Olly Robertson, James Shearer, Adrienne Shum, Petr Slovak, Polly Waite, Edmund J. S. Sonuga-Barke

**Affiliations:** 1 Institute of Psychiatry, Psychology & Neuroscience, King’s College London, London, UK; 2 Departments of Psychiatry and Experimental Psychology, University of Oxford, Oxford, UK

**Keywords:** COVID-19, Randomised controlled trial, Protocol, Child behaviour problems, Parenting, Intervention, Digital health

## Abstract

**Objectives:**

The COVID-19 related lockdowns and distancing measures have presented families with unprecedented challenges. A UK-wide cohort study tracking changes in families’ mental health since early lockdown (Co-SPACE) found a significant rise in primary school-aged children’s behaviour problems and associated family-related stress. Three-quarters of parents in Co-SPACE also reported wanting extra support. In SPARKLE, we will examine whether providing Co-SPACE families with a smartphone application delivering information and parenting support, *Parent Positive,* can reverse the negative effects of the pandemic on children and parents. The efficacy on child and parent outcomes and cost-effectiveness of *Parent Positive* will be examined. We will also test whether the effects are moderated by pre-existing levels of child conduct problems and usage of *Parent Positive*. Exploratory analyses will examine whether other baseline characteristics or lockdown circumstances moderate the effects of *Parent Positive*.

**Trial design:**

SPARKLE is a two-arm superiority parallel group randomised controlled trial embedded in an existing large UK-wide self-selected community cohort – Co-SPACE. Those who consent to SPARKLE will be randomised 1:1 to either *Parent Positive* or Follow-up As Usual (FAU).

**Participants:**

Co-SPACE (a UK-wide longitudinal cohort study) parents aged ≥18 who have children aged 4-10 years will be eligible for SPARKLE.

**Intervention and comparator:**

***Parent Positive:*** is a digital public health intervention that can be delivered rapidly at scale to support parents in managing their children’s behaviour to reduce conduct problems and levels of family conflict, which were exacerbated during the first lockdown, and which may increase further in future months as families need to cope with continuous uncertainty and further disruption to their daily lives. Co-designed with parents and based on decades of parenting research, *Parent Positive* consists of three elements: (i) Parenting Boosters: where advice, delivered in the form of narrated animations, videos, graphics and text is provided to help parents with eight common parenting challenges; (ii) Parenting Exchange: a facilitated parent-to-parent communication and peer support platform and; (iii) Parent Resources: giving access to carefully selected high-quality, evidence-based online parenting resources.

***Follow-up as Usual:*** FAU was selected as a comparator because the public health nature meant that an active comparator was not appropriate due to the pragmatic, rapid implementation of the trial. Individuals randomised to FAU will receive no intervention for the first two months while the data for baseline (T1), T2 and T3 are collected. They will then be given full access to the app until 30th November 2021.

**Main outcomes:**

Outcome measures will be collected remotely through Qualtrics according to the Co-SPACE schedule at baseline (T1), which will be the Co-SPACE survey data obtained immediately prior to randomisation, and then at one month (T2) and two months (T3) post-randomisation. Measures will be collected to assess group differences in child and parent outcomes, costs and service utilisation, and adverse events. Usage of Parent Positive will also be tracked. The primary outcome is parent-reported child conduct problems at one-month post-randomisation measured using the Strengths and Difficulties Questionnaire conduct problems subscale.

**Randomisation:**

Enrolled participants will be allocated to *Parent Positive* or FAU at the ratio of 1:1 by simple randomisation using the Randomizer function within the Qualtrics programme. Neither blocking nor stratification will be used.

**Blinding (masking):**

It is not possible to blind parents enrolled in the study and Qualtrics will automatically inform parents of their group allocation. Blinded members of the research team and the senior statistician will not be given access to the Qualtrics system or the data in order to remain blinded until after the analysis is complete. We do not anticipate any serious harms associated with taking part in the intervention, therefore there will be no need to unblind any blinded staff during the study. The junior statistician will be unblinded throughout.

**Numbers to be randomised (sample size):**

A total of 616 will be recruited into the trial with 308 consenting parents randomised to each treatment arm.

**Trial status:**

V1.0; 15.03.2021. Not yet recruiting. Anticipated start date: 1^st^ April 2021. Anticipated end date for recruitment: 31^st^ July 2021.

**Trial registration:**

Clinicaltrial.gov: NCT04786080. The trial was prospectively registered on 8 March 2021.

**Full protocol:**

The full protocol is attached as an additional file, accessible from the Trials website (Additional file 1). In the interest in expediting dissemination of this material, the familiar formatting has been eliminated; this Letter serves as a summary of the key elements of the full protocol. The study protocol has been reported in accordance with the Standard Protocol Items: Recommendations for Clinical Interventional Trials (SPIRIT) guidelines (Additional file 2).

**Supplementary Information:**

The online version contains supplementary material available at 10.1186/s13063-021-05226-4.

## Supplementary Information

**Additional file 1.** Full Study Protocol.

**Additional file 2.** SPIRIT 2013 Checklist: Recommended items to address in a clinical trial protocol and related documents.

## Data Availability

On completion of the study a clean, anonymised data set will be made available for open access via the UK Data Service or another suitable repository.

