# Supplementary material for: Supporting Parents & Kids Through Lockdown Experiences (SPARKLE): A digital parenting support app implemented in an ongoing general population cohort study during the COVID-19 pandemic: A structured summary of a study protocol for a randomised controlled trial
Source: Trials. 2021 Apr 10;22:267. doi: 10.1186/s13063-021-05226-4 (PMC8035596; doi:10.1186/s13063-021-05226-4)
Supplement: Supplementary file 1 — Additional file 1. Full Study Protocol. [file 13063_2021_5226_MOESM1_ESM.docx]

1. **Title:** *Supporting Parents & Kids Through Lockdown Experiences (SPARKLE)*: A randomised controlled trial of a digital parenting support app implemented in an ongoing general population cohort study during the COVID-19 pandemic.
2. **Trial identifier and registry name.** The trial was prospectively registered on Clinicaltrials.gov: NCT04786080
3. **Date and version identifier:** SPARKLE version 1.0: 15.03.2021. This protocol follows the SPIRIT reporting guidelines (Chan et al., 2013).
4. **Funding source:** SPARKLE: Grant ES/V016393/1 from the Economic and Social Research Council, UK. Co-SPACE: Grant 2004CQ002/BS3 from the UKRI and Grant from the Westminster Foundation.
5. **a) Names, affiliations, and roles of protocol contributors**

Katarzyna Kostyrka-Allchorne (Co-I). Department of Child & Adolescent Psychiatry, Institute of Psychiatry, Psychology & Neuroscience, King’s College London ([kasia.kostyrka-allchorne@kcl.ac.uk](mailto:kasia.kostyrka-allchorne@kcl.ac.uk)).

Cathy Creswell (Co-I). Departments of Psychiatry and Experimental Psychology, University of Oxford ([cathy.creswell@psych.ox.ac.uk](mailto:cathy.creswell@psych.ox.ac.uk)).

Sarah Byford (Co-I – Senior Trial Health Economist). Department of Health Service and Population Research, Institute of Psychiatry, Psychology & Neuroscience, King’s College London ([sarah.byford@kcl.ac.uk](mailto:sarah.byford@kcl.ac.uk)).

Crispin Day (Clinical collaborator). Department of Psychology, Institute of Psychiatry, Psychology & Neuroscience, King’s College London ([crispin.1.day@kcl.ac.uk](mailto:crispin.1.day@kcl.ac.uk)).

Kimberley Goldsmith (Co-I – Senior Trial Statistician). Department of Biostatistics and Health Informatics, Institute of Psychiatry, Psychology & Neuroscience, King’s College London ([kimberley.goldsmith@kcl.ac.uk](mailto:kimberley.goldsmith@kcl.ac.uk)).

Marta Koch (SPARKLE Research Administrator). Department of Child & Adolescent Psychiatry, Institute of Psychiatry, Psychology & Neuroscience, King’s College London ([marta.koch@kcl.ac.uk](mailto:marta.koch@kcl.ac.uk)).

Walter Muruet Gutierrez (Junior Trial Statistician). Department of Biostatistics and Health Informatics, Institute of Psychiatry, Psychology & Neuroscience, King’s College London ([walter.muruet_gutierrez@kcl.ac.uk](mailto:walter.muruet_gutierrez@kcl.ac.uk)).

Melanie Palmer (SPARKLE Research Fellow). Department of Child & Adolescent Psychiatry, Institute of Psychiatry, Psychology & Neuroscience, King’s College London ([melanie.palmer@kcl.ac.uk](mailto:melanie.palmer@kcl.ac.uk)).

Jasmine Raw (SPARKLE Research Assistant). Departments of Psychiatry and Experimental Psychology, University of Oxford ([Jasmine.Raw@pgr.reading.ac.uk](mailto:Jasmine.Raw@pgr.reading.ac.uk)).

Olly Robertson (SPARKLE Research Assistant). Departments of Psychiatry and Experimental Psychology, University of Oxford (olly.robertson@psych.ox.ac.uk).

James Shearer (Junior Trial Health Economist). Department of Health Service and Population Research, Institute of Psychiatry, Psychology & Neuroscience, King’s College London ([james.shearer@kcl.ac.uk](mailto:james.shearer@kcl.ac.uk)).

Adrienne Shum (Co-SPACE Research Assistant). Departments of Psychiatry and Experimental Psychology, University of Oxford ([hei.shum@psych.ox.ac.uk](mailto:hei.shum@psych.ox.ac.uk)).

Petr Slovak (HCI collaborator). Department of Child & Adolescent Psychiatry, Institute of Psychiatry, Psychology & Neuroscience, King’s College London ([petr.slovac@kcl.ac.uk](mailto:petr.slovac@kcl.ac.uk)).

Polly Waite (Co-I). Departments of Psychiatry and Experimental Psychology, University of Oxford ([polly.waite@psych.ox.ac.uk](mailto:polly.waite@psych.ox.ac.uk)).

Edmund J S Sonuga-Barke (PI - corresponding author). Department of Child & Adolescent Psychiatry, Institute of Psychiatry, Psychology & Neuroscience, King’s College London ([edmund.sonuga-barke@kcl.ac.uk](mailto:edmund.sonuga-barke@kcl.ac.uk)).

1. **b) Name and contact information for the trial sponsor**

King’s College London

Address: Room 5.31, James Clerk Maxwell Building, 57 Waterloo Road, London SE1 8WA

Telephone: +44 (0)207 8483224

Email: [reza.razavi@kcl.ac.uk](mailto:reza.razavi@kcl.ac.uk)

1. **c) Role of sponsor in trial design**

The sponsors and funders played no role in the trial design.

1. **d) Trial oversight**

A Trial Steering Committee (TSC) will be convened and will meet approximately every 4 months throughout the study. Recruitment to the trial will be rapid and no interim analyses are planned so a separate Data Monitoring and Ethics Committee (DMC) will not be formed. However, we reserve the option to form one if the TSC deem it necessary at any point during the trial.

**Protocol amendments**

The following changes have been made to the protocol.

| *Version* | *Date* | *Amendment* |
| --- | --- | --- |
|  |  |  |

1. **Introduction**

The COVID-19 related lockdowns and continuing distancing measures have presented families with unprecedented challenges. Extended joint confinement, often within very limited space, isolation from friends and family, increased demands on parents to deliver home-schooling in face of competing work demands, mental health- and money-related worries, all have placed relationships between parents and their children under great pressure. Consistent with this, Co-SPACE, a UK-wide, UKRI and Westminster Foundation-funded cohort study tracking changes in families’ mental health since early lockdown (Waite & Creswell, 2020) found a significant rise in parent-reported children’s behaviour problems and associated family-related stress (Skripkauskaite et al. 2021; Shum et al., 2021). Strikingly, 70% of parents in Co-SPACE reported wanting extra support concerning their child’s response to the pandemic and associated restrictions (Waite, Patalay, Moltrecht, McElroy, & Creswell, 2020).

There is also widespread concern amongst professionals that these problems may increase further as the pandemic continues, and with it, the restrictions put on people’s lives. Families are faced with the continuous need to readjust to new routines, structures and challenges. There is also ongoing disruption to school attendance. Between September and December 2020 many children in the UK were facing periods of repeated isolation due to cases being identified among staff and pupils. Since January 2021 and the introduction of a third national lockdown, schools switched to remote teaching for a second time and for the majority of children parents were again required to supervise remote learning or even deliver home-schooling. These continuous challenges are going to increase pressures on schools (Henshaw, 2020) and already overstretched children’s services (Ford, 2020), as more parents seek additional support and advice from professionals. This will further increase the already substantial gap between provision and need, leaving many families without access to vital support and unresolved difficulties that are more likely to become persistent and impairing. To address this, public health level interventions are needed to support parents in managing common difficulties and stressors. Parenting interventions, based on behavioural and social learning theories, are recommended as the first approach for intervention across a range of child mental health and behavioural difficulties (e.g., National Institute for Health and Care Excellence, 2013).

In SPARKLE, we will examine whether providing families of primary school-aged children within the Co-SPACE cohort with a digital application delivering information and parenting support, *Parent Positive,* can reverse the negative effects of the pandemic on children’s conduct problems while reducing levels of psychological distress in the parents. *Parent Positive* is a digital public health intervention that can be delivered rapidly at scale to support parents in managing their children’s behaviour to reduce conduct problems and levels of family conflict, which were exacerbated during the first lockdown, and which may increase further in future months as families need to cope with continuous uncertainty and further disruption to their daily lives. Co-designed with parents and based on decades of parenting research, *Parent Positive* consists of three elements: (i) *Parenting Boosters:* where advice, delivered in the form of narrated animations, videos, graphics and text is provided to help parents with eight common parenting challenges; (ii) *Parenting Exchange*: a facilitated parent-to-parent communication and peer support platform and; (iii) *Parent Resources:* giving access to carefully selected high-quality, evidence-based online parenting resources. As such, SPARKLE represents one of the first attempts to embed a parenting intervention trial into an ongoing longitudinal cohort study as part of a rapid deployment strategy for intervening to mitigate the negative effects of a national health crisis on families. By doing this, it has potential to provide the evidence base to motivate the national dissemination of *Parent Positive.*

1. **Objectives**

SPARKLE has six aims:

1. To test whether *Parent Positive* reduces the average levels of parent-reported child conduct problems in a self-selected community sample, measured over a one (primary outcome) and two month (secondary outcome) period, when compared to Follow-up As Usual (FAU).
2. To test whether *Parent Positive* reduces the average levels of parent psychological distress, parental child-related stress and worries, family conflict and child emotional problems (additional secondary outcomes) in a self-selected community sample, measured over a one and two month period, when compared to FAU.
3. To assess the extent to which the effects of *Parent Positive* on the primary conduct outcome are moderated by levels of (i) pre-existing levels of conduct problems and (ii) *Parent Positive* usage levels as monitored during the one month intervention period. Usage of *Parent Positive* will be monitored for two months for descriptive purposes.
4. To test the cost-effectiveness of *Parent Positive* compared to FAU over a two month follow-up period.
5. **Exploratory:** To assess whether the effects of *Parent Positive* on primary and secondary outcomes are moderated by baseline characteristics - family social economic status and composition, parental psychological distress, child age, symptoms of attention-deficit/hyperactivity disorder and emotional problems.
6. **Exploratory:** (ii) to explore the impact of lockdown circumstances and policies at baseline on the effects of *Parent Positive* on primary and secondary outcomes. Data on lockdown circumstances will be collected at one and two months post-randomisation for descriptive purposes.

Note that these exploratory aims may not be addressed in the primary trial results paper.

Hypotheses:

1. At both one and two months post-randomisation, *Parent Positive* will reduce the average levels of parent-reported child conduct problems in a self-selected community sample, when compared to FAU.
2. *Parent Positive* will reduce the average levels of parent psychological distress, parental child-related stress and worries, family conflict and child emotional problems (secondary outcomes) in comparison to FAU.
3. The effects on the primary outcome will be greater where (i) children had higher baseline levels of conduct problems (i.e., more need for *Parent Positive*) and (ii) parents spent more time using the app.
4. *Parent Positive* will be cost-effective compared to FAU as a result of improved outcomes and cost savings.
5. **Trial design**

SPARKLE is a two-arm superiority parallel group RCT embedded in an existing large self-selected community cohort – Co-SPACE. Co-SPACE parents/carers (hereafter referred to as ‘parent’ for simplicity) aged ≥18 who have children aged 4-10 years will be invited to take part in the SPARKLE trial. Those who consent to SPARKLE will be randomised 1:1 to either *Parent Positive* or FAU. Outcome measures will be collected according to the Co-SPACE schedule at baseline (T1), which will be the Co-SPACE survey data obtained immediately prior to randomisation, and then at one month (T2) and two months (T3) post-randomisation. After completion of T3, parents will continue to be involved in Co-SPACE according to the Co-SPACE data collection schedule unless consent is withdrawn.

1. **Study setting**

SPARKLE will be embedded in Co-SPACE, a UK-wide longitudinal cohort study of mental health of parents, children and adolescents (4-16 years) during the COVID-19 pandemic (for more detailed information see Waite & Creswell, 2020, http://cospaceoxford.org/). The study started in March 2020 and has been tracking families every month using Qualtrics – an online data collection platform. From April 2021 to July 2021 an invitation to SPARKLE will be embedded in the Co-SPACE survey sent to parents of children aged 4-10 years.

1. **Inclusion & exclusion criteria**

First, the following Co-SPACE inclusion criteria will apply: parent is willing and able to give informed consent, must be at least 18 years old and lives in the UK. There are no exclusion criteria for Co-SPACE. Second, SPARKLE-specific criteria will be: parent has a child aged 4-10 years and has access to a smartphone with operating system OS 8-9 or higher (Android devices) or iOS 12-13 or higher (Apple devices). There will be no further exclusion criteria. Children will not be selected based on pre-existing conduct problems.

1. **Interventions**
   1. **Parent Positive:** This is an app developed for use on a smartphone providing a flexible digital space where parents can get support and advice to help them manage their children’s behaviour. It was developed by Sonuga-Barke, Kostyrka-Allchorne, Palmer, Day, Robertson, Koch and Slovak with digital design partner [TOAD](https://www.toadlondon.com/).

It consists of three zones:

1. The *Parenting Boosters* zone includes structured advice, support and tips for parents to deal with eight common parenting challenges. These are based around the series of [Families Under Pressure](https://familiesunderpressure.maudsleycharity.org/) animations identified and developed through co-design with parents. To increase their attractiveness and reach these were scripted to be light-hearted, humorous and non-judgmental and are delivered by eight high-profile British celebrities who are also parents. The eight messages relate to: (i) staying positive and motivated (Olivia Colman); (ii) making sure everyone knows what is expected of them (Sharon Horgan); (iii) building your child’s self-confidence and trust (Danny Dyer); (iv) getting your child to follow instructions (Rob Brydon); (v) promoting better behaviour (Jessica Ennis-Hill); (vi) limiting conflict (Holly Willoughby); (vii) keeping calm when your kids act up (Romesh Ranganathan); and (viii) careful use of sanctions (Shappi Khorsandi). In *Parent Positive,* the animations are supplemented by extended content to provide more information and advice for each challenge drawing on the [Empowering Parents, Empowering Communities](http://www.cpcs.org.uk/index.php?page=empowering-parents-empowering-communities) resources and the [STEPS](https://www.kcl.ac.uk/research/online-parent-training-for-the-initial-management-of-adhd-referral-optima) resources delivered in the form of videos, graphics and text – all based on over 20 years evidence from parent training studies (Daley et al. 2018).
2. The *Parenting Exchange* zone provides a facilitated parent-to-parent communication platform where parents are encouraged to raise any specific challenges they are facing to receive support from other parents. Trained parent facilitators will moderate the exchange and create posts to enhance engagement with the intervention. The Exchange will be also used to collate questions that parents have for experts (e.g., developmental psychologists, parent training practitioners, nutritionists, etc.) about a range of topics related to both the *Parent Positive* challenges and broader issues of direct relevance to children’s behaviour (e.g., sleep, diet, etc). These questions will be answered by experts at pre-recorded webinars held regularly during the period of the trial. The recording will be made available to parents using the app.
3. The *Parent Resources* zone will provide links to carefully selected high-quality, evidence-based online parenting resources reviewed and approved by a committee of parenting experts.

The app will be free and parents will be able to access the information when needed and in the order they choose. Parents in the intervention group will receive access during the immediate post-randomisation period until 30^th^ November 2021. To access the app, parents will receive an automated email with a link to download it from either Google Play (for Android users) or the App Store (for Apple users) together with brief instructions on how to download the app on the smartphone and register as a user.

- 1. **Follow-up as Usual (FAU):** FAU was selected as a comparator because the public health nature meant that an active comparator was not appropriate due to the pragmatic, rapid deployment of the trial. Individuals randomised to FAU will receive no intervention for the first 10 weeks while the data for baseline (T1), T2 and T3 are collected. They will then be given full access to the app until 30^th^ November 2021.
  2. **Criteria for discontinuing intervention.** We do not expect using *Parent Positive* to lead to harm that would suggest the intervention needs to be discontinued. However, parents will be able to withdraw from the trial at any point for any reason.
  3. **Strategies to increase engagement with the app and levels of app usage.** The traditional notion of adherence to an intervention protocol does not apply to public health intervention trials where samples are not restricted to those with a specific clinical need. This is the case in SPARKLE where we expect that a significant proportion of parents who are enrolled in the trial may not feel they need to use the app (i.e., those not having difficulties parenting their children’s behaviour). Nevertheless, we will initially encourage parents to use the app and promote high levels of engagement using push notifications on their mobile phone reminding them to engage with *Parent Positive*. Including the Parenting Exchange in the app is also expected to improve engagement. First, participants will be able to communicate with each other by posting and reading messages. Second, they will receive social feedback from their peers on their posts made within the Exchange. Third, they will be able to ask questions to experts via the Exchange.
  4. **Permitted concomitant care.** There will be no restrictions on concomitant care which will be monitored through collection of information on service use and harms.

1. **Measures and Outcomes**

[Co-SPACE](http://cospaceoxford.org/) routinely collects a wide range of measures. In this protocol we only describe those measures that will be included in the SPARKLE analysis. See Table 1 for the schedule of data collection for each measure. Where there are specific instructions for dealing with missing data for each measure, these will be followed. Where there are no specific instructions for missing data, we will prorate at the subscale level where there are 20% or fewer missing subscale items. For measures with no subscales, we will prorate at the overall scale level where there are 20% or fewer missing items overall.

- 1. **Child outcomes**
     1. **Conduct problems (T1 co-variate; primary outcome T2; secondary outcome T3; T1 moderator)**: This will be measured using the parent-rated conduct problems subscale of the Strengths and Difficulties Questionnaire (SDQ; Goodman, 1997). This is a widely used and validated five item subscale measuring oppositional, defiant and disruptive behaviour rated on a 3-point Likert scale (not true, somewhat true, and certainly true), with a mix of positive and negatively phrased items. Individual items’ scores are summed to derive an overall symptom subscale score. The level of ‘caseness’ at baseline will be presented for descriptive purposes. Data from this measure have been routinely collected from the Co-SPACE cohort every month.

Child conduct problems score was selected as the primary outcome because of the Co-SPACE finding reported above of a significant increase in average levels in the community since early on in the UK pandemic.

**Table 1: Schedule of enrolment, interventions and assessment**

|  | | | **STUDY PERIOD** | | | |
| --- | --- | --- | --- | --- | --- | --- |
|  | | | **Baseline** | **Intervention** | **Follow-up**  **1** | **Follow-up**  **2** |
| **Timepoint** | | | **1** |  | **2** | **3** |
| **Month** | | | 0 |  | 1 | 2 |
| **Sample description** | | |  |  |  |  |
|  | Family characteristics and demographics | | X |  |  |  |
| **Outcomes** | | |  |  |  |  |
|  | Child | SDQ conduct problems | X |  | X | X |
|  |  | SDQ emotional problems | X |  | X | X |
|  | Parent | Parental child-related stress and worries | X |  | X | X |
|  |  | Family conflict | X |  | X | X |
|  |  | DASS-21 psychological distress | X |  | X | X |
| **Other measures** | | |  |  |  |  |
|  | SDQ ADHD symptoms | | X |  | X | X |
|  | SDQ peer problems | | X |  | X | X |
|  | SDQ prosocial behaviour | | X |  | X | X |
|  | CA-SUS service utilisation | |  |  | X | X |
|  | Lockdown circumstances | | X |  | X | X |
|  | Self-reported adverse events | |  |  | X | X |
| **Interventions randomised 1:1** | | |  |  |  |  |
|  | *Parent Positive* | |  | Access to app | | |
|  | Follow-up As Usual^a^ | |  | No access to app | | |
| **Intervention usage and acceptability** | | |  |  |  |  |
|  | Total time spent accessing the *Parenting Boosters* | |  |  | X | X |
|  | Other app usage metrics | |  |  | X | X |
|  | Parent Positive Attitudes | |  |  | X | X |
| *Note.* CA-SUS=Child and Adolescent Service Use Schedule; DASS-21=Depression, Anxiety & Stress Scale-21; SDQ=Strengths and Difficulties Questionnaire.  ^a^=Parents allocated to Follow-up As Usual will get access to Parent Positive after the Follow-up 2 (T3) assessment. | | | | | | |

- - 1. **Emotional problems (T1 co-variate, T2 and T3 secondary outcome; T1 moderator):** measured by the respective SDQ subscale (Goodman, 1997). This five-item subscale measures fearfulness, anxiety and low mood rated on a 3-point Likert scale (not true, somewhat true, and certainly true) with positively phrased items. Individual items’ scores are summed to derive an overall symptoms subscale score.
  1. **Parent/family outcome**
     1. **Parental child-related stress and worries levels (T1 co-variate; T2 and T3 secondary outcomes):** This is based on five items routinely measured in Co-SPACE regarding children’s behaviour, wellbeing, screen time use, education and future using a 4-point Likert scale (not at all, a little, quite a lot, a great deal). Individual questions’ scores are summed to generate a single score. The scale has an adequate level of internal consistency (Cronbach alpha > .70) and re-test reliability (*r* = .71) amongst Co-SPACE parents.
     2. **Family conflict (T1 co-variate; T2 and T3 secondary outcomes):** This is based on three items routinely collected in Co-SPACE relating to arguments between parents, parents and children and siblings which are measured on a 4-point Likert scale (not at all, a bit, a lot, completely). The three questions are summed to generate a total score. The scale has acceptable internal consistency (Cronbach alpha > .54) and test-retest reliability (*r* = .73) amongst Co-SPACE parents.
     3. **Parental psychological distress (T1 co-variate; T2 and T3 secondary outcome; T1 moderator):** The Depression, Anxiety and Stress Scales-21 (DASS-21) is a widely validated 21-item self-report questionnaire measuring adult depression, anxiety and stress (Lovibond & Lovibond, 1995). Each of these three subscales consists of seven items rated on a 4-point Likert scale (did not apply to me at all; applied to me to some degree, or some of the time; applied to me to a considerable degree, or a good part of the time, applied to me very much, or most of the time), which are summed to obtain a single subscale score. The subscales scores will be summed and multiplied by two to form a single psychological distress measure comparable to the DASS-42 measure.
  2. **Other measures**
     1. **ADHD symptoms (T1 moderator; T1, T2 and T3 for QALYs calculation)**: measured with the inattention/overactivity subscale of the SDQ (Goodman, 1997). This five-item subscale measures restlessness, overactivity and inattention rated on 3-point Likert scales (not true, somewhat true, and certainly true), with a mix of positive and negatively phrased items. Individual items’ scores are summed to derive an overall symptoms subscale score.
     2. **Peer problems (T1, T2 and T3 for QALYs calculation)**: measured with the peer problems subscale of the SDQ (Goodman, 1997). This five-item subscale measures difficulties in peer relationships and bullying rated on 3-point Likert scales (not true, somewhat true, and certainly true), with a mix of positive and negatively phrased items. Individual items’ scores are summed to derive an overall peer problems subscale score.
     3. **Prosocial behaviour (T1, T2 and T3 for QALYs calculation)**: measured with the prosocial behaviour subscale of the SDQ (Goodman, 1997). This five-item subscale measures difficulties in peer relationships and bullying rated on 3-point Likert scales (not true, somewhat true, and certainly true), with positively phrased items. Individual items’ scores are summed to derive an overall prosocial subscale score with higher scores indicating more prosocial behaviour.
     4. **Attitudes to Parent Positive (T2 and T3, intervention arm only):** Parent mean ratings of *Parent Positive* using a questionnaire, consisting of three items measuring parents’ ratings of the usefulness of each individual zone rated on a 7-point scale (1 = not useful at all to 7 = very useful), which will be summed up to derive an overall app usefulness score. The questionnaire will also capture parents’ descriptive and qualitative views of *Parent Positive* – both positive and negative.
     5. **Estimate of resource use for the health economic analysis (T2 and T3):** Information about the child’s use over the two-month follow-up period of key health and social services (high cost, high volume) known from published studies to be of relevance to the current population will be collected using a modified version of the Child and Adolescent Service Use Schedule (CA-SUS; Barrett et al., 2012). The CA-SUS will be completed by the parent at T2 and T3, covering the previous month. This measure has been developed and successfully applied in a range of populations of young people with mental health conditions, including self-complete versions, and will be based primarily on an online, self-completed parent-reported version of the CA-SUS designed for use in a current, related study (the NIHR funded OPTIMA trial - On-Line Parent Training for the Initial Management of ADHD referrals; RP-PG-0618-20003).
     6. **App usage data (During intervention period, intervention arm only):** Usage data will be broken down by *Parent Positive* elements (challenges) and domains (Boosters, Exchange, Resources). This will allow descriptive analysis of usage patterns. The analysis of the impact of usage levels on the effects of *Parent Positive* (see analysis section below) variables will be based on a derived measure of total time accessing the “Boosters” zone during the one month intervening period. Other app usage metrics will be collected (e.g., engagement in Exchange zone).
     7. **Family characteristics and demographic measures (T1 co-variates; sample description):** Parents will provide information about their child’s age and gender, their own and their child’s ethnicity, number of children in the family, number of rooms in the family home and access to outside space. This information will be used to describe the sample and an overcrowding index will be calculated by dividing the number of individuals in the household by the number of rooms. We will estimate family SES based on total household income, coded as < £16,000 | £16,000 - £29,999 | £30,000 - £59,999 | £60,000 - £89,999 | £90,000 - £119,999 | > £120,000 | Prefer not to say, likely collapsing small categories.
     8. **COVID-19 pandemic lockdown circumstances (T1 moderator; T2 descriptives; T3 descriptives):** Parents will provide information on the pandemic-related restrictions they are currently facing. This will include details of the current lockdown policy in their local area (partial or full lockdown), whether parents are working from home, and whether children are being home-schooled.

1. **Time schedule of enrolment and assessment**

Recruitment for the trial will be between April to July 2021. Final T3 assessments will be concluded in September 2021. The enrolment and group allocation procedure is shown in Figure 1. All parents aged 18+ of children aged 4-10 years completing the Co-SPACE survey between April and July 2021 will receive an invitation to take part in SPARKLE – this will appear at the beginning of the Qualtrics survey. Parents who express interest in taking part will be provided with an information sheet about the SPARKLE trial and those who wish to take part will be provided with an online consent form to be completed within Qualtrics. Prior to consenting, parents will be asked to confirm that they have access to a compatible smartphone. Consenting parents will be randomised within Qualtrics using a Randomizer tool and followed-up using a separate survey branch. Qualtrics will automatically inform the parent about their group allocation. A researcher responsible for providing participants with instructions regarding the app access will obtain information about the outcome of randomisation from the Qualtrics database.


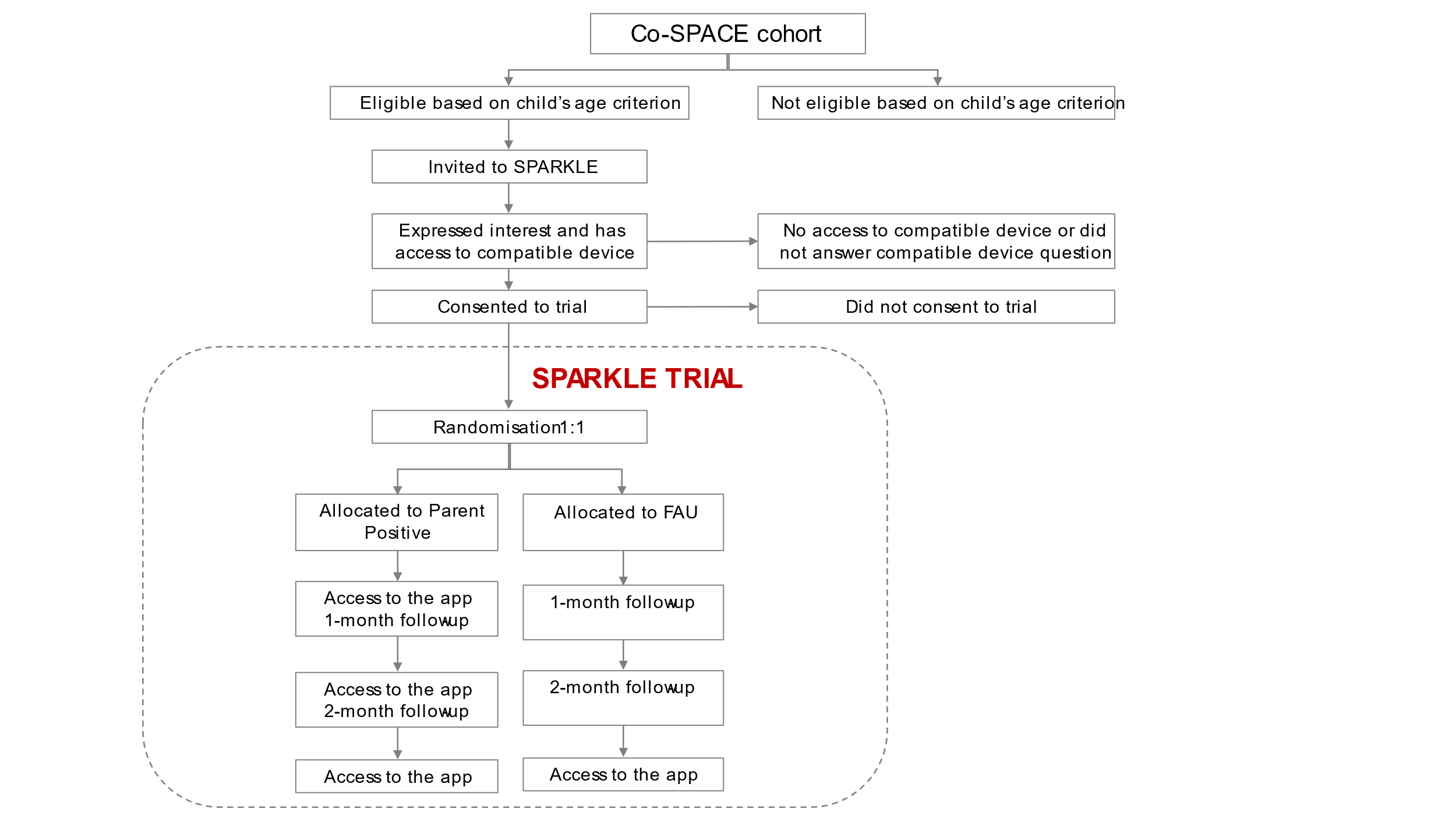


**Figure 1. SPARKLE enrolment and group allocation procedure.**

1. **Sample**

A total of 616 will be recruited into the trial with 308 consenting parents randomised to each arm following SPARKLE baseline assessments. This sample size was powered to address our primary question whether comparing outcomes at one month post-randomisation, *Parent Positive* will reduce children’s conduct problems observed as compared to FAU. During the first UK lockdown (i.e., March to June 2020) there was around an approximate 0.2 standard deviation increase in SDQ conduct problems scores reported by Co-SPACE parents with children aged 4-10. For the current study, we took this as representing the smallest between group difference that was of clinical value to detect between *Parent Positive* and FAU scores post-randomisation (i.e., Cohen’s *d* of 0.2). We assumed a within trial drop-out rate of 30%, a correlation of 0.5 between one pre- and two post-randomisation measures (Machin, Campbell, Tan, & Tan, 2018), and using a one-tailed (Parent Positive > FAU) and an alpha of .05 that this number of participants provides 90% power to test the one-sided hypothesis that *Parent Positive* is superior to FAU.

1. **Recruitment**

Participants will be recruited into SPARKLE from the pool of Co-SPACE families of children aged 4-10. Recruitment into the Co-SPACE study is ongoing using advertisements on social media and through circulating information about the study to primary schools and relevant organisations, so SPARKLE may also recruit participants that are new to Co-SPACE.

1. **Allocation**

Participants will be enrolled into the study online via the Quatrics platform. Following consenting, simple randomisation will be carried out remotely within the Qualtrics programme using the Randomizer function, after which the parents in the *Parent Positive* group will be given access to the app as in section 7.4 above. Enrolled participants will be allocated to *Parent Positive* or FAU at the ratio of 1:1 by simple randomisation. Neither blocking nor stratification will be used.

1. **Blinding**

It is not possible to blind parents enrolled in the study and Qualtrics will automatically inform parents of their group allocation. Data will be collected remotely by Qualtrics. Blinded members of the research team and the senior statistician will not be given access to the Qualtrics system or the data in order to remain blinded until after the analysis is complete. We do not anticipate any serious harms associated with taking part in the intervention, therefore there will be no need to unblind any blinded staff during the study. The junior statistician will be unblinded throughout.

1. **Data collection**

SPARKLE data will be collected at monthly intervals at three time points following the Co-SPACE schedule as indicated above (Baseline [T1], T2, and T3) using the Qualtrics platform. Finally, in SPARKLE, *Parent Positive* usage data will be collected automatically within the app across the intervention period as described above.

1. **Data management**

Handling of the data collected in Co-SPACE is described in detail in the Co-SPACE Data Management Plan. The SPARKLE Data Management Plan outlines how SPARKLE-specific data collected outside of the Co-SPACE routine follow-ups will be managed and stored during and after the project is completed. The junior statistician will request periodic data extracts from the Co-SPACE (baseline) and SPARKLE (follow-up) teams; these will be shared via a restricted OneDrive for Business link, then moved to King’s College SharePoint location, which is a web-based, Microsoft Office integrated collaborative platform. The data provided from Co-SPACE will be linked to the *Parent Positive* usage data using the SPARKLE ID. The data stored on SharePoint are encrypted and access to the data will be restricted only to those who need to have it.

1. **Statistical analysis**

**Aim i) Does *Parent Positive* reduce the average levels of parent-reported child conduct problems in a self-selected community sample, measured over a one (primary outcome) and two month (secondary outcome) period, when compared to Follow-up As Usual (FAU)?**

**Aim ii) Does *Parent Positive* reduce the average levels of parent psychological distress, parental child-related stress and worries, family conflict and child emotional problems (additional secondary outcomes) in a self-selected community sample, measured over a one and two month period, when compared to FAU**?

Briefly, a mixed effects linear analysis of covariance (ANCOVA) model with repeated measures will be used with **the SDQ conduct problem** scores at one (primary outcome) and two months (secondary outcomes) post-randomisation as the dependent variables, and intervention group, time point, intervention group by time point interaction, baseline SDQ conduct problem score, gender and age of child as independent variables, with the interaction term used to extract the primary and secondary outcome effects at the two time points. Similar statistical models will be used to test for intervention effects on the other secondary outcome scale scores (average levels of parent psychological distress, parental child-related stress and worries, family conflict and child emotional problems) at one and two months post-randomisation. All analysis will follow the intention to treat (ITT) principle as far as possible (Sullivan et al., 2018; White et al., 2012).

**Aim iii) Are the effects of *Parent Positive* on the primary conduct problem outcome** **moderated by levels of (i) pre-existing levels of conduct problems (subgroup analysis) and (ii) *Parent Positive* usage levels as monitored during the one month intervention period (post-randomisation intervention** **effect modification analysis).**

As for the concept of adherence, the concept of per protocol analysis seems less sensible in this trial where the intervention is taking more of a public health approach with no specific direction of how and when to use, in a sample not restricted to those with a specific clinical need. In this sense, and for this particular intervention, there is not a “intervention protocol” that parents need to adhere to. However, an analysis of the extent to which effects depend on (i) pre-existing levels of child conduct problems and (ii) intervention uptake will help to interpret the value of the app. To address (i) in the mixed model for conduct problems described under Aims i and ii, the intervention by time interaction term will be replaced with an intervention by time by baseline conduct problems interaction. Intervention effects for different conduct problem scores will be extracted at the two time points. To address (ii) the effect of the app usage measure, total time accessing the Boosters zone during the one month intervening period, will be assessed using appropriate causal methods for post-randomisation variables measured only in the Parent Positive group (Dunn et al., 2015). Child gender and age will be entered as independent variables, as well as other variables that could be predictors of app usage (i.e., pre-existing levels of conduct problems). Usage of *Parent Positive* will be monitored for two months for descriptive purposes.

**Aim iv) Is *Parent Positive* cost-effective compared to FAU over a two month follow-up period.**

The primary economic evaluation will be a cost-utility analysis carried out two months post-randomisation and taking the National Health Service (NHS) and personal social services perspective preferred by National Institute for Health and Care Excellence (NICE), including any education-based health and social services, given the age of the population. Using data from the CA-SUS, all services will be costed using nationally applicable unit costs (e.g., Personal Social Services Research Unit Costs of Health and Social Care, NHS Reference Costs, British National Formulary for medications). The *Parent Positive* costs will be estimated in consultation with intervention developers.

At both time points (one and two months post-randomisation), utility weights for children will be derived from the SDQ using a published mapping algorithm (Furber, Segal, Leach, & Cocks, 2014) that transforms SDQ subscale scores into Child Health Utility 9 Dimensions (CHU-9D; Stevens, 2010) scores which can then be used to estimate quality adjusted life years (QALYs) between baseline and two month follow-up.

Costs and outcomes will be compared in terms of mean differences and 95% confidence intervals from non-parametric bootstrap regressions (1,000 replications) to account for non-normal distribution common to economic data. Cost-effectiveness will be assessed using the net benefit approach following standard approaches (Drummond, Sculpher, Claxton, Stoddart, & Torrance, 2015). A joint distribution of incremental mean costs and effects for the two groups will be generated using non-parametric bootstrapping to explore the probability that each of the intervention conditions is the optimal choice, subject to a range of possible maximum values (ceiling ratio) that a decision-maker might be willing to pay for improvements in outcome (QALYs). Cost-effectiveness will be explored using incremental cost effectiveness ratios (Briggs, 1999) with uncertainty represented by cost-effectiveness planes and cost-effectiveness acceptability curves (Fenwick, Claxton, & Sculpher, 2001).

**Aim v and vi) Exploratory – note these analyses may not be included in the primary trial results paper.**

**(v) Are the effects of *Parent Positive* on primary and secondary outcomes moderated by other baseline characteristics - parental psychological distress and child age, gender, ADHD symptoms and emotional problems**?

**(vi) Are the effects of *Parent Positive* on primary and secondary outcomes moderated by baseline lockdown-related circumstances – full vs. partial or no lockdown, whether parents are working from home or not, and whether children are being homeschooled or not.**

Similar models to those described for Aim iii will be used to explore whether i) these additional baseline characteristic variables and ii) lockdown-related circumstances moderate intervention effects on the outcomes. Child gender and age will be retained as independent variables in all models for consistency.

1. **Data Monitoring**

Given the short-term nature of the trial and rapid recruitment schedule, there is little opportunity to monitor data during the trial. As noted, the trial will have an independent TSC, but no DMC. The TSC will include an independent statistician and will be consulted as to whether they want any data reporting to be done during the trial. This will be included in the TSC Terms of Reference that will be agreed upon by the Committee. Given the duration of the trial and the nature of the intervention, there are no interim analysis or stopping rules either for efficacy or safety.

1. **Harms**

There are no known risks or harms associated with completing the intervention. However, parents will be informed through the information sheet that there is some possibility that using *Parent Positive* may result in them having new concerns about their child’s behaviour, their relationship with their child or their ability to manage as a parent. They will also be informed that this is the reason why we will ask them to report any physical or psychological difficulties during the study (see Adverse events). They will also be advised to contact their General Practitioner if they feel they need further support.

*Definitions*

Adverse event (AE): Any untoward medical occurrence in a clinical trial participant administered a research product or procedure which does not necessarily have a causal relationship with this treatment. Medical adverse events will be recorded using standard medical definitions.

Serious adverse event (SAE): Any AE, which results in death, is life-threatening, requires hospitalisation or prolonged hospitalisation, causes persistent or significant disability or incapacity, or consists of a congenital abnormality or birth defect. Important medical events that may not be immediately life-threatening or result in death or hospitalisation but may jeopardise the patient or may require intervention to prevent one of the other outcomes listed in the definition above should also be considered serious.

Psychological events: Self-reported emergence or a substantial increase in detrimental behaviour or circumstance including: child behaviour, child participation in school or extra-curricular activities, parent psychological distress, family conflict, and child protection concerns. Parents will also be asked whether they have experienced any other negative/positive events that they feel are important to their participation in the trial.

Adverse events will be asked about at T2 and T3 and reported retrospectively covering the previous month. All reported events will be reviewed by the research team to identify any SAEs. All SAEs will be recorded and closely monitored until resolution, stabilisation, or until it has been shown that the study intervention is not the cause.

1. **Auditing**

Auditing is not applicable to this protocol.

1. **Ethics**

All relevant study documents have been reviewed and approved by the King’s College London – Psychiatry, Nursing and Midwifery Research Ethics Subcommittee, reference number HR-20/21-21451 and the University of Oxford Central University Research Ethics Committee, reference numbers R73153/RE001. Any future protocol amendments will be documented and submitted for ethical approval prior to implementation. No recruitment activity took place before ethical approval for the study had been obtained. Co-SPACE ethical approval was granted by the University of Oxford Central University Research Ethics Committee, reference numbers R69060/RE001.

1. **Protocol amendments**

In case of new information becoming available, which may result in significant changes to the risks and benefits of taking part, the PIS and informed consent form will be reviewed and updated accordingly. All parents actively enrolled in the study will be informed of the updated information and will be given a revised copy of the PIS and informed consent form to confirm their wish to continue taking part.

1. **Consent**

Invitation to take part in SPARKLE will appear at the beginning of the routinely completed Co-SPACE survey. All Co-SPACE parents will have already given consent to take part in Co-SPACE through Qualtrics. Those who respond to the invitation to participate in SPARKLE will be re-directed to SPARKLE's online Participant Information Sheet (PIS) within Qualtrics for more information. The PIS will explain the study procedures and will provide a contact for a researcher available to answer any questions that the parent has concerning the study. Parents will also be informed that participation is completely voluntary, and they are under no obligation to take part and they can withdraw at any time without giving a reason. Research data can be withdrawn from analysis by contacting the research team through email until 30^th^ September 2021. Before a parent enters a study, they will be asked to complete the informed consent form provided electronically within Qualtrics. A copy of the completed consent form will be emailed to the parent and a record of the consent will be retained in the electronic study files.

1. **Confidentiality**

SPARKLE will require access to administrative data (i.e., email address, mobile phone number) to enable participants to access the *Parent Positive* app and to notify them about the updates*. Parent Positive* will collect information about device IDs and IP addresses, which will be used to track app usage. The full list of other information collected by the app can be found [here](https://aws.amazon.com/compliance/data-privacy/).

The PI and all members of the research team will take every effort to preserve the confidentiality of parents taking part in the study. To de-identify the data, each participant name will be replaced by an assigned a SPARKLE study ID linked to the Co-SPACE ID. A reference file with the study IDs and participant identifiable information will be kept on a secure, restricted-access folder on the University of Oxford server and will be backed up on an external encrypted hard drive.

Participant’s identifiable data required for administrative purposes (e.g. name and contact details) will be stored in a separate file from the pseudonymised data files. These will be accessed only by those members of the research team who are responsible for contacting parents (e.g., to email a link to the online survey). No individual participant’s data will be identifiable in the publications or reports that may result from this study. Personal information will be stored for 36 months in conformity to GDPR.

1. **Financial and other competing interests**

ESB, KK, MP, CD, OR, MK and PS developed the Parent Positive with digital design partner [TOAD](https://www.toadlondon.com/). ESB has received speaker fees, consultancy or research funding from Takeda, Neurotech Solutions, QBtech and Medice. He has received royalties from the New Forest Parenting Programme.

KG’s contributions represent independent research part funded by the NIHR Biomedical Research Centre (South London and Maudsley NHS Foundation Trust and King’s College London) and the NIHR Applied Research Collaboration South London (King’s College Hospital NHS Foundation Trust). The views expressed are those of the authors and not necessarily those of the NHS, the NIHR, the Department of Health and Social Care or The Stroke Association.

1. **Access to data**

On completion of the study a clean, fully anonymised data set will be made available for open access via the UK Data Service or another suitable repository. Questionnaire and app data will be shared along with a procedures document, curation document and description of variables. Links to the data record will be provided in all papers published using the data.

1. **Post-trial care**

After completion of T3, parents in both intervention groups will have access to the app until 30^th^ November 2021. During this time we will also email parents once a month with a short reminder about other organisations that are available to support families.

1. **Dissemination of trial results**

A full and complete account of the research will be published in a high-quality peer reviewed journal. In addition, the findings will be disseminated through oral and poster presentations at a range of interdisciplinary events and seminars organised within the team's collaborative network. There will also be a general dissemination programme for parents of primary school-aged children and the general public.

If results are positive, Parent Positive will be disseminated rapidly through collaboration with Public Health England and the Department for Education, in cooperation with commercial media partners. The app requires no additional costly personal support or direction by clinical or educational staff and can be directly deployed across multiple settings (schools, clinics, etc.) by different professional groups without extra training. It will run on most mobile phones and will not rely on continuous access to the Internet, thus making it accessible to parents living in areas with poor connectivity.

**References**

Antony, M. M., Bieling, P. J., Cox, B. J., Enns, M. W., & Swinson, R. P. (1998). Psychometric properties of the 42-item and 21-item versions of the Depression Anxiety Stress Scales in clinical groups and a community sample. *Psychological assessment, 10*(2), 176.

Barrett, B., Byford, S., Sharac, J., Hudry, K., Leadbitter, K., Temple, K., . . . consortium, P. (2012). Service and wider societal costs of very young children with autism in the UK. *Journal of autism and developmental disorders, 42*(5), 797-804.

Briggs, A. H. (1999). A Bayesian approach to stochastic cost‐effectiveness analysis. *Health economics, 8*(3), 257-261.

Chan, A. W., Tetzlaff, J. M., Altman, D. G., Laupacis, A., Gotzsche, P. C., Krle A-Jeric, K., Hrobjartsson, A., Mann, H., Dickersin, K., Berlin, J. A., Dore, C. J., Parulekar, W. R., Summerskill, W. S., Groves, T., Schulz, K. F., Sox, H. C., Rockhold, F. W., Rennie, D., & Moher, D. (2013). SPIRIT 2013 statement: Defining standard protocol items for clinical trials.*Annuals of Internal Medicine, 38*(6), 506-514. S1020-49892015001100011

Daley, D., Van Der Oord, S., Ferrin, M., Cortese, S., Danckaerts, M., Doepfner, M., Van den Hoofdakker, B.J., Coghill, D., Thompson, M., Asherson, P., Banaschewski, T., Brandeis, D., Buitelaar, J., Dittmann, R.W., Hollis, C., Holtmann, M., Konofal, E., Lecendreux, M., Rothenberger, A., Santosh, P., Simonoff, E., Soutullo, C., Steinhausen, H.C., Stringaris, A., Taylor, E., Wong, I.C., Zuddas, A. and Sonuga‐Barke, E.J. (2018), Practitioner Review: Current best practice in the use of parent training and other behavioural interventions in the treatment of children and adolescents with attention deficit hyperactivity disorder. *Journal of Child Psychology and Psychiatry,* *59*: 932-947.

Drummond, M. F., Sculpher, M. J., Claxton, K., Stoddart, G. L., & Torrance, G. W. (2015). *Methods for the economic evaluation of health care programmes*: Oxford university press.

Dunn, G., Emsley, R., Liu, H., Landau, S., Green, J., White, I., & Pickles, A. (2015). Evaluation and validation of social and psychological markers in randomised trials of complex interventions in mental health: A methodological research programme.*Health Technology Assessment (Winchester, England), 19*(93), 1-vi.

Fenwick, E., Claxton, K., & Sculpher, M. (2001). Representing uncertainty: the role of cost‐effectiveness acceptability curves. *Health economics, 10*(8), 779-787.

Ford, T. (2020). Challenges for children’s services as Lockdown eases. Retrieved from <https://reachwell.org/2020/07/16/prof-tamsin-ford-challenges-for-childrens-services-as-lockdown-eases/>

Furber, G., Segal, L., Leach, M., & Cocks, J. (2014). Mapping scores from the Strengths and Difficulties Questionnaire (SDQ) to preference-based utility values. *Quality of Life Research, 23*(2), 403-411.

Goodman, R. (1997). The Strengths and Difficulties Questionnaire: a research note. *Journal of Child Psychology and Psychiatry, 38*(5), 581-586.

Henshaw, P. (2020). Exclusion warning as schools braced for 'fresh behavioural challenges' after lockdown. Retrieved from <https://www.headteacher-update.com/news/exclusion-warning-as-schools-braced-for-fresh-behavioural-challenges-after-lockdown/226794>

Lancet, T. (2020). Redefining vulnerability in the era of COVID-19. *Lancet (London, England), 395*(10230), 1089.

Lovibond, S. H., & Lovibond, P. F. (1995). *Manual for the Depression Anxiety & Stress Scales* (2nd ed.). Sydney: Psychology Foundation.

Machin, D., Campbell, M. J., Tan, S. B., & Tan, S. H. (2018). *Sample sizes for clinical, laboratory and epidemiology studies*: John Wiley & Sons.

McElroy, E., Patalay, P., Moltrecht, B., Shevlin, M., Shum, A., Creswell, C., & Waite, P. (2020). Demographic and health factors associated with pandemic anxiety in the context of COVID-19.

National Institute for Health and Clinical Excellence. (2013). *Antisocial behaviour and conduct disorders in children and young people: Recognition and management (CG158).* Manchester: National Institute for Health and Clinical Excellence.

Pearcey, S., Shum, A., Waite, P., Patalay, P., & Creswell, C. (2020). *Report 05: Changes in children and young people’s mental health symptoms and ‘caseness’ during lockdown and patterns associated with key demographic factors*. Retrieved from <https://cospaceoxford.org/wp-content/uploads/2020/08/Co-SPACE-report-05-SDQ-symptoms-caseness-demographics_29-10-20.pdf>

Shum, A., Skripkauskaite, S., Pearcey, S., Raw, J., Waite, P., & Creswell C. (2021). Report 07: Changes in parents’ mental health symptoms and stressors from April to December 2020. Retrieved from <https://cospaceoxford.org/wp-content/uploads/2021/01/Report_07_19JAN.pdf>

Skripkauskaite, S., Shum, A., Pearcey, S., Raw, J., Waite, P., & Creswell C. (2021). *Report 08: Changes in children’s and young people’s mental health symptoms: March 2020 to January 2021.* Retrieved from [https://cospaceoxford.org/wp-content/uploads/2021/02/Report_08_17.02.21.pdf](https://cospaceoxford.org/wp-content/uploads/2021/02/Report_08_17.02.21.pdf%20)

Stevens, K. J. (2010). Working with children to develop dimensions for a preference-based, generic, pediatric, health-related quality-of-life measure. *Qualitative Health Research, 20*(3), 340-351.

Sullivan, T. R., White, I. R., Salter, A. B., Ryan, P., & Lee, K. J. (2018). Should multiple imputation be the method of choice for handling missing data in randomized trials?*Statistical Methods in Medical Research, 27*(9), 2610-2626. 10.1177/0962280216683570

Waite, P., & Creswell, C. (2020). *Protocol: Covid-19: Supporting Parents, Adolescents and Children during Epidemics – Co-SPACE*.

Waite, P., Patalay, P., Moltrecht, B., McElroy, E., & Creswell, C. (2020). *Report 02: Covid-19 worries, parent/carer stress and support needs, by child special educational needs and parent/carer work status*. Retrieved from <https://cospaceoxford.org/wp-content/uploads/2020/07/Co-SPACE-report-02.pdf>

White, I. R., Carpenter, J., & Horton, N. J. (2012). Including all individuals is not enough: Lessons for intention-to-treat analysis.*Clinical Trials, 9*(4), 396-407.

**Table 2. Table showing key aspects of the SPARKLE trial**

| Data category | Information |
| --- | --- |
| Primary registry and trial identifying number | Clinicaltrials.gov: NCT04786080 |
| Date of registration in primary registry | 08/03/2021 |
| Secondary identifying numbers | - |
| Source(s) of monetary or material support | The Economic and Social Research Council, UK. Grant number: ES/V016393/1 |
| Primary sponsor | Kings’ College London |
| Secondary sponsor(s) | N/A |
| Contact for public queries | Professor Edmund Sonuga-Barke |
| Contact for scientific queries | Professor Edmund Sonuga-Barke |
| Public title | *Supporting Parents & Kids Through Lockdown Experiences* (SPARKLE) |
| Scientific title | *Supporting Parents & Kids Through Lockdown Experiences (SPARKLE)*: A randomised controlled trial of a digital parenting support app implemented in an ongoing general population cohort study during the COVID-19 pandemic |
| Countries of recruitment | United Kingdom |
| Health condition(s) or problem(s) studied | Behavioural problems, mental health |
| Intervention(s) | Parent Positive |
| Key inclusion and exclusion criteria | Aged 18 or over |
|  | Parent of a child aged 4-10 |
|  | Access to a compatible digital device |
| Study type | Two-arm superiority parallel group randomised controlled trial |
| Date of first enrolment | April 2021 (anticipated) |
| Target sample size | 616 |
| Recruitment status | Not started yet |
| Primary outcome(s) | Mean child conduct problems (SDQ) |
| Key secondary outcomes | Mean child emotional problems (SDQ)  Mean parental child-related stress and worries  Mean family conflict  Total parental psychological distress (DASS-21)  *Other measures*  Family characteristics and demographics  Mean child ADHD (SDQ)  Mean child peer problems (SDQ)  Mean child prosocial behaviour (SDQ)  Service utilisation (CA-SUS)  Lockdown circumstances  Adverse events  App usage metrics  Parent Positive attitudes |
